# Lateral cortical *Cdca7* expression levels are regulated by Pax6 and influence the production of intermediate progenitors

**DOI:** 10.1186/s12868-017-0365-0

**Published:** 2017-06-05

**Authors:** Yu-Ting Huang, John O. Mason, David J. Price

**Affiliations:** Centre for Integrative Physiology, University of Edinburgh, Hugh Robson Building, George Square, Edinburgh, EH8 9XD UK

**Keywords:** Cortical development, Intermediate progenitors, Proliferation, In utero electroporation, Pax6, Gradients, Cdca7, Neurogenesis

## Abstract

**Background:**

We studied whether regulation of *Cdca7* (*Cell division cycle associated 7*) expression by transcription factor Pax6 contributes to Pax6’s cellular actions during corticogenesis. The function of Cdca7 in mediating Pax6’s effects during corticogenesis has not been explored. Pax6 is expressed by radial glial progenitors in the ventricular zone of the embryonic cortical neuroepithelium, where it is required for the development of a normal complement of Tbr2-expressing intermediate progenitor cells in the subventricular zone. Pax6’s expression levels are graded across the ventricular zone, with highest levels laterally where Tbr2-expressing progenitors are generated in greatest numbers at early stages of corticogenesis.

**Methods:**

We used in situ hybridization and immunohistochemistry to analyse patterns of *Cdca7* and *Pax6* expression in cortical tissue from wild-type and *Pax6* ^−*/*−^ embryos. In each genotype we compared the graded expression of the two genes quantitatively at several ages. To test whether defects in Cdca7 expression in lateral cortical cells might contribute to the cellular defects in this region caused by Pax6 loss, we electroporated a Cdca7 expression vector into wild-type lateral cortex and examined the effect on the production of Tbr2-expressing cells.

**Results:**

We found that *Cdca7* is co-expressed with *Pax6* in cortical progenitors, at levels opposite to those of *Pax6*. Lowest levels of *Cdca7* are found in the radial glial progenitors of lateral cortex, where Pax6 levels are highest. Higher levels of Cdca7 are found in ventral telencephalon, where Pax6 levels are low. Loss of Pax6 causes *Cdca7* expression to increase in the lateral cortex. Elevating Cdca7 in normal lateral cortical progenitors to levels close to those normally found in ventral telencephalon reduces their production of Tbr2-expressing cells early in lateral cortical formation.

**Conclusion:**

Our results suggest that Pax6 normally represses *Cdca7* expression in the lateral cortex and that repression of *Cdca7* in cells of this region is required for their production of a normal complement of Tbr2-expressing intermediate progenitors.

## Background

In rodents, about two thirds of excitatory, pyramidal neurons in the cerebral cortex are generated from progenitor cells that divide beneath the apical surface of the embryonic cortical neuroepithelium in a region called the subventricular zone [1–3]. These cells, known as intermediate progenitor cells, are marked by their expression of the transcription factor Tbr2 [4]. Most of them divide just once to generate two postmitotic neurons that migrate into the developing cortical plate (the future cortex) [2, 5, 6]. During this process, these differentiating cells activate expression of the transcription factor Tbr1 [4]. Intermediate progenitors are themselves generated by the division of radial glial cells at the apical (luminal) surface of the neuroepithelium, in the ventricular zone [7–9]. Radial glial cells express the transcription factor Pax6, intermediate progenitors and postmitotic neurons do not [10].

Early in corticogenesis, Pax6 expression levels are graded across the ventricular zone of the developing cortical neuroepithelium. Levels increase with proximity to the boundary between the lateral cortex and the subpallium. Pax6 exerts regional control of cell proliferation, with its greatest effects in anterior and lateral regions where its levels are highest [11]. It is required for the development of a normal complement of Tbr2-expressing intermediate progenitors [12]. The mechanisms by which it regulates these processes are poorly understood.

Previous work screening for transcriptional changes in the cortex of mice with a null-mutation of *Pax6* identified *Cdca7* (*Cell division cycle associated 7*) as a gene whose expression is increased by loss of Pax6 function [11]. It is a nuclear protein containing a zinc finger DNA-binding domain in both human and mouse [13]. Most of what we know about the functions of Cdca7 comes from studies in non-cortical systems. In the haematopoietic system there is evidence that it affects cell specification and differentiation rather than proliferation [14]. Elsewhere, it has been associated with neoplastic transformation [15, 16], but its mechanisms of action are unclear. It is a target of the E2F family of transcription factors, which are involved in cell proliferation and whose activity is regulated by Pax6 in the developing cortex [11, 13].

In the current work, we first analysed the relationship between the normal expression patterns of *Pax6* and *Cdca7* and the effects of Pax6 loss on *Cdca7* expression. We found evidence that Pax6 represses *Cdca7* expression in the lateral cortex, where Pax6 levels are normally highest, with loss of Pax6 increasing lateral cortical expression of *Cdca7*. We then studied the effects of increasing Cdca7 expression in the lateral cortex by electroporating a Cdca7 expression vector into this region. We found that elevated Cdca7 levels reduce the production of Tbr2+ and Tbr1+ cells early in lateral cortical formation, suggesting that repression of Cdca7 by Pax6 is required for the normal production of intermediate progenitors in this region.

## Methods

### Mice

**Fig. 1:**
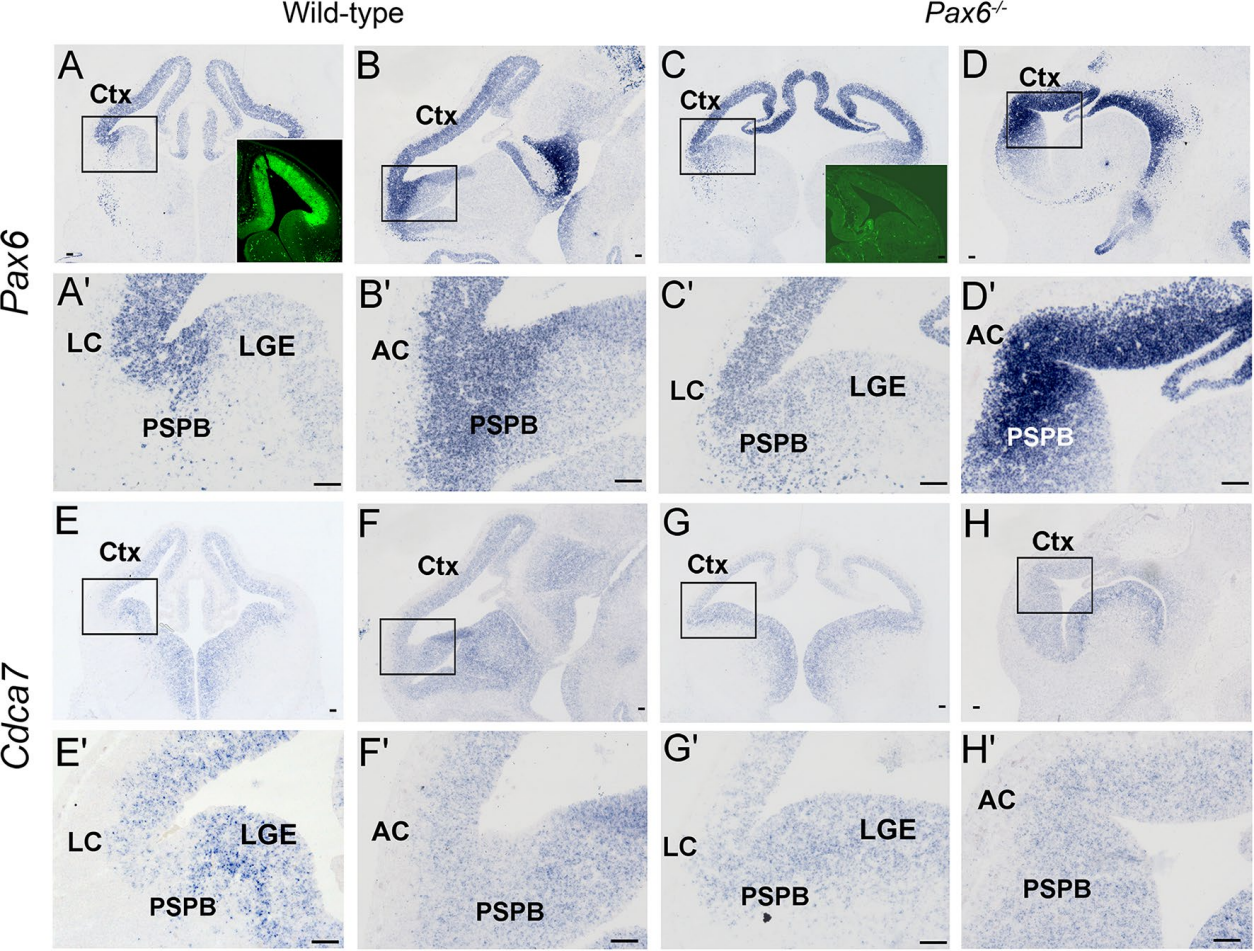
Expression of *Pax6* and *Cdca7* at E12.5 in wild-type and *Pax6* ^−*/*−^ telencephalon. In situ hybridizations on coronal (**A**, **A**′, **C**, **C**′, **E**, **E**′, **G**, **G**′) and sagittal sections (**B**, **B**′, **D**, **D**′, **F**, **F**′, **H**, **H**′). **A**, **A**′, **B**, **B**′ *Pax6* expression is graded in the cortex (Ctx) from high rostro-laterally to low caudo-medially with highest expression level at the lateral cortex (LC) in **A**, **A**′ and the anterior cortex (AC) in **B**, **B**′ and a sharp reduction at the pallial-subpallial boundary (PSPB) in wild-types. *Inset* in **A** shows immunohistochemistry for Pax6 protein in wild-type at E12.5. LGE, lateral ganglionic eminence. **A**′–**B**′ High magnification of areas outlined in **A**, **B**. **C**, **C**′, **D**, **D**′ *Pax6* expression is abnormal in the cortex and decreases less sharply at the PSPB in *Pax6* ^−*/*−^ embryos. *Inset* is **C** shows immunohistochemistry on E12.5 *Pax6* ^−*/*−^ telencephalon; although *Pax6* mRNA is produced, there is no detectable protein. **C**′–**D**′ High magnification of areas outlined in **C**, **D**. **E**, **E**′, **F**, **F**′ The telencephalic *Cdca7* expression pattern in the wild-type appears opposite to that of *Pax6*, with low levels on the cortical side of the PSPB, in the lateral cortex. **E**′, **F**′ High magnification of areas outlined in **E**, **F**. **G**, **G**′, **H**, **H**′ *Cdca7* expression is abnormal in the cortex and shows no obvious decrease on the cortical side of the PSPB in *Pax6* ^−*/*−^ embryos. **G**′, **H**′ High magnification of the areas outlined in **G**, **H**. *Scale bars* 50 µm

*Pax6* ^−*/*−^ embryos were obtained by crossing *Pax6* ^*Sey/*+^ heterozygous mice [17]. In the *Pax6* ^*Sey*^ allele, a point mutation has introduced a premature stop codon between the DNA binding elements; mRNA is produced but is unable to generate functional protein [17] (and see insets in Fig. 1A, C). All mice were maintained on an albino CD1 background. The morning of vaginal plug appearance was designated embryonic day 0.5 (E0.5). All procedures were approved by Edinburgh University’s Animal Ethics Committee and were done under licence in accordance with regulations contained in the UK Home Office Animals (Scientific Procedures) Act 1986.

### In situ hybridisation

Embryos were fixed overnight in 4% paraformaldehyde, cryoprotected with 30% sucrose and sectioned at 10 μm. In situ hybridization followed a standard protocol [18]. Antisense RNA probes were labelled with digoxigenin (DIG, Roche). To make plasmids for *Cdca7* probe generation, the following primers were used: *Cdca7* forward, TCTGAGAGCTCTGCAAACGA; *Cdca7* reverse, AGCTGCAGTTGCAAATTCCT. A 745 bp *Cdca7* fragment was cloned into pGEM-T easy vector (Promega). This was linearized with SacII and the probe was generated using SP6 RNA polymerase. The generation of probe for *Pax6* was described previously [19].

### Immunohistochemistry

Cryosections (10 μm thick) were subject to antigen retrieval by microwaving for 20 min in 10 mM sodium citrate buffer. After blocking with 20% donkey serum, sections were incubated with primary antibodies overnight at 4 °C. The primary antibodies used were mouse anti-Pax6 (Developmental Studies Hybridoma Bank 1:100), rabbit anti-Cdca7 (Proteintech 1:100), goat anti-GFP and rat anti-BrdU (5-Bromo-2′-deoxyuridine, 1:200 Abcam), rabbit anti-Tbr1 and rabbit anti-Tbr2 (Abcam 1:100), 4′,6-Diamidino-2-Phenylindole (DAPI, Invitrogen 1:1000). Secondary antibodies were conjugated with Alexa Fluor 488 or 568 (Invitrogen). Fluorescent and confocal images were acquired with a Leica DM5500B microscope and a Nikon A1R confocal microscope, respectively.

### Generation of Cdca7-expression vector

*Cdca7* cDNA open reading frame (ORF) obtained from Origene Technologies (clone MG205975; NCBI accession number NM_025866) was combined with sequence for an influenza hemagglutinin (HA) epitope at its 3′ end to generate a HA-tagged *Cdca7* ORF (Fig. 5B). NheI restriction sites at both 3′ and 5′ ends were used to clone the tagged *Cdca7* cDNA into the expression plasmid pCAGGS_IRES-NLS-GFP (called pCAGGS_GFP) [20]. The Cdca7 expression vector is called pCAGGS_Cdca7.

### Cell culture, transfection and western blotting

Human Embryonic Kidney (HEK) 293 cells were maintained in DMEM medium supplemented with 10% foetal bovine serum. Cells were incubated at 37 °C with 5% CO_2_ and transfected using Lipofectamine 2000 (Invitrogen). Cell lysates were collected with Passive Lysis Buffer (Promega) 48 h post transfection. For western blotting, the primary antibodies were rabbit anti-HA (Abcam 1:1000) and mouse anti-Glyceraldehyde 3-phosphate dehydrogenase (GAPDH, Ambion 1:5000).

### In utero electroporation

Wild-type CD1 females were used for in utero electroporation. Purified plasmid DNA (pCAGGS_GFP control or pCAGGS_Cdca7) was adjusted to 2 μg/μl. In the afternoon of the electroporation day, animals were given one dose of analgesia (Buprenorphine, 0.05 mg/kg, subcutaneously) and received isoflurane throughout the operation. Plasmids (approximately 2 μg) were injected into the lateral ventricle of embryos aged E12.5 or E14.5. Electroporation was achieved using CUY21 (Sonidel Limited), 32 Volts for E12.5 and 35 Volts for E14.5 embryos in 50 ms pulses with 950 ms intervals. All procedures were covered by Home Office Project Licence 60/4545 and personal licence 60/13925, following the on-site regulations and were approved by the Edinburgh University veterinary physicians. A single dose of bromodeoxyuridine (BrdU, Sigma) in 0.9% NaCl was administrated intraperitoneally to the pregnant females (70 μg/g of body weight) 15.5 h post-electroporation. Pregnant females were sacrificed 30 min after BrdU injection and embryos were used in histological procedures described above.

### Quantifications

To estimate gradients of expression, average values (±SEM) for slopes of intensity plots were calculated from 4 wild-type and 4 *Pax6* ^−*/*−^ embryos at E12.5 and at E14.5 and 3 embryos of each genotype at E13.5 (Fig. 3). To estimate frequencies of Tbr2+ and Tbr1+ and BrdU+ cells in the population of GFP+ cells after E12.5 electroporation we used 4 control and 3 experimental embryos, and after E14.5 electroporation we used 3 control and 4 experimental embryos. Each embryo was from a different litter. Images were acquired using a Nikon A1R confocal microscope. Proportions of GFP+ cells that were either Tbr2+, Tbr1+ or BrdU+ were counted in sampling regions up to 200 µm wide in 3 sections through the core of the electroporated region in each embryo. To estimate relative levels of endogenously and exogenously driven Cdca7 protein expression, fluorescence intensity values were obtained by outlining cells of interested and measuring each one’s total grey intensity using Fiji software. Average values under different conditions were obtained from multiple cells. All comparisons were done within the same images to avoid effects of batch and differential exposure.

## Results

### Comparison of *Pax6* and *Cdca7* expression patterns in wild-type and *Pax6*^−*/*−^ embryos

Pax6 is expressed at high levels in the ventricular zone of the developing cerebral cortex and at lower levels in the ventricular zone of the lateral ganglionic eminence (LGE). At the earliest stages of murine cortical neurogenesis, around embryonic days 12–13 (E12-13), *Pax6* expression levels are graded from rostro-lateral [high] to caudo-medial [low]. As corticogenesis continues, this gradient of *Pax6* expression becomes progressively less steep [11]. At all embryonic ages, Pax6 expression levels drop sharply at the boundary between pallium and subpallium. We tested whether the expression pattern of *Cdca7* normally correlates with that of *Pax6* and whether the expression of *Cdca7* is affected in mutants that are null for *Pax6*.

**Fig. 2:**
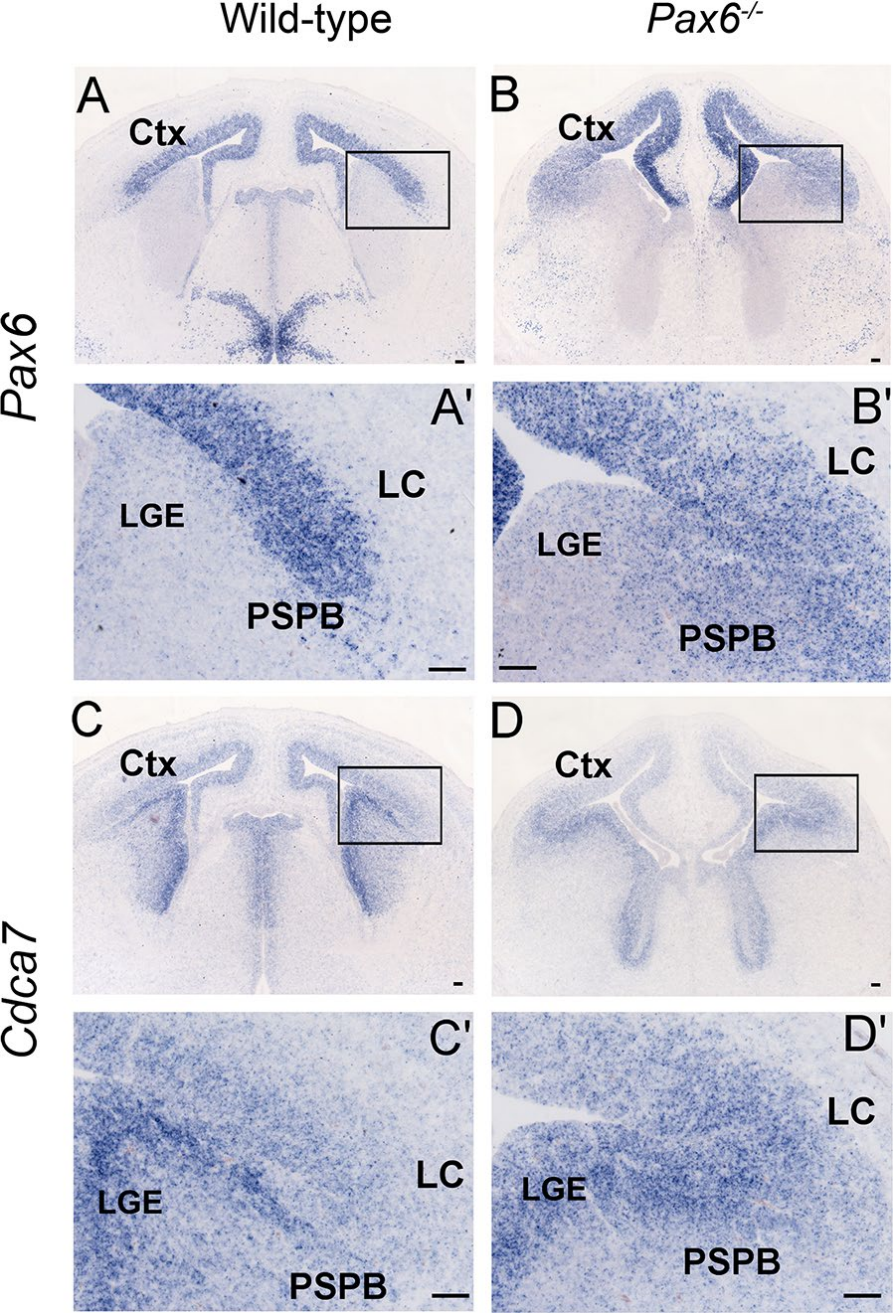
Expression of *Pax6* and *Cdca7* at E14.5 in wild-type and *Pax6* ^−*/*−^ telencephalon. In situ hybridizations on coronal sections. **A**, **A**′, **B**, **B**′ *Pax6* expression declines sharply at the PSPB in wild-type telencephalon and more gradually in *Pax6* ^−*/*−^ telencephalon. **A**′, **B**′ High magnification of areas outlined in **A**, **B**. **C**, **C**′, **D**, **D**′ *Cdca7* expression declines sharply on the pallial side of the PSPB in wild-types but not in *Pax6* ^−*/*−^ embryos. **C**′, **D**′ High magnification of areas outlined in **C**, **D**. *Scale bars* 50 µm

In situ hybridization was used to reveal the expression patterns of *Pax6* and *Cdca7* in adjacent sections from wild-type (WT; n = 4) and *Pax6* ^−*/*−^ (n = 4) embryos at E12.5 (Fig. 1). Note that while the *Pax6* null mutants used here do not produce functional Pax6 protein (insets in Fig. 1A, C), they do still express mutant *Pax6* mRNA [21, 22]. The expression pattern of *Pax6* described above can be seen in Fig. 1A, A′, B, B′. There were much higher levels of *Pax6* in lateral cortex than in LGE, with a sharp transition at the pallial-subpallial boundary (PSPB). In contrast, *Cdca7* expression levels were lower in the lateral cortex than in the LGE (Fig. 1E, E′, F, F′). In E12.5 *Pax6* ^−*/*−^ embryos, the decline in *Pax6* expression across the PSPB was more gradual than normal (Fig. 1C, C′, D, D′) and the dip in *Cdca7* expression levels in the region of high *Pax6* expression in the lateral cortex was no longer obvious (Fig. 1G, G′, H, H′). By E14.5, wild-type *Pax6* expression levels were consistently high across the pallium and, as at E12.5, they showed a sharp decline at the PSPB (Fig. 2A, A′; n = 4). Wild-type *Cdca7* expression levels again showed an opposing pattern, being lower in the lateral cortex than in the LGE with a sharp change in levels at the PSPB (Fig. 2C, C′). In E14.5 *Pax6* ^−*/*−^ embryos (n = 4), *Pax6* expression declined more gradually than normal across the PSPB, as at E12.5 (Fig. 2B, B′), but the high levels of *Cdca7* expression observed in subpallium continued across the PSPB deep into the lateral cortex where they now overlapped high *Pax6* expression (Fig. 2D, D′). These findings suggest that Pax6 is a negative regulator of *Cdca7* in the lateral cortex.

**Fig. 3:**
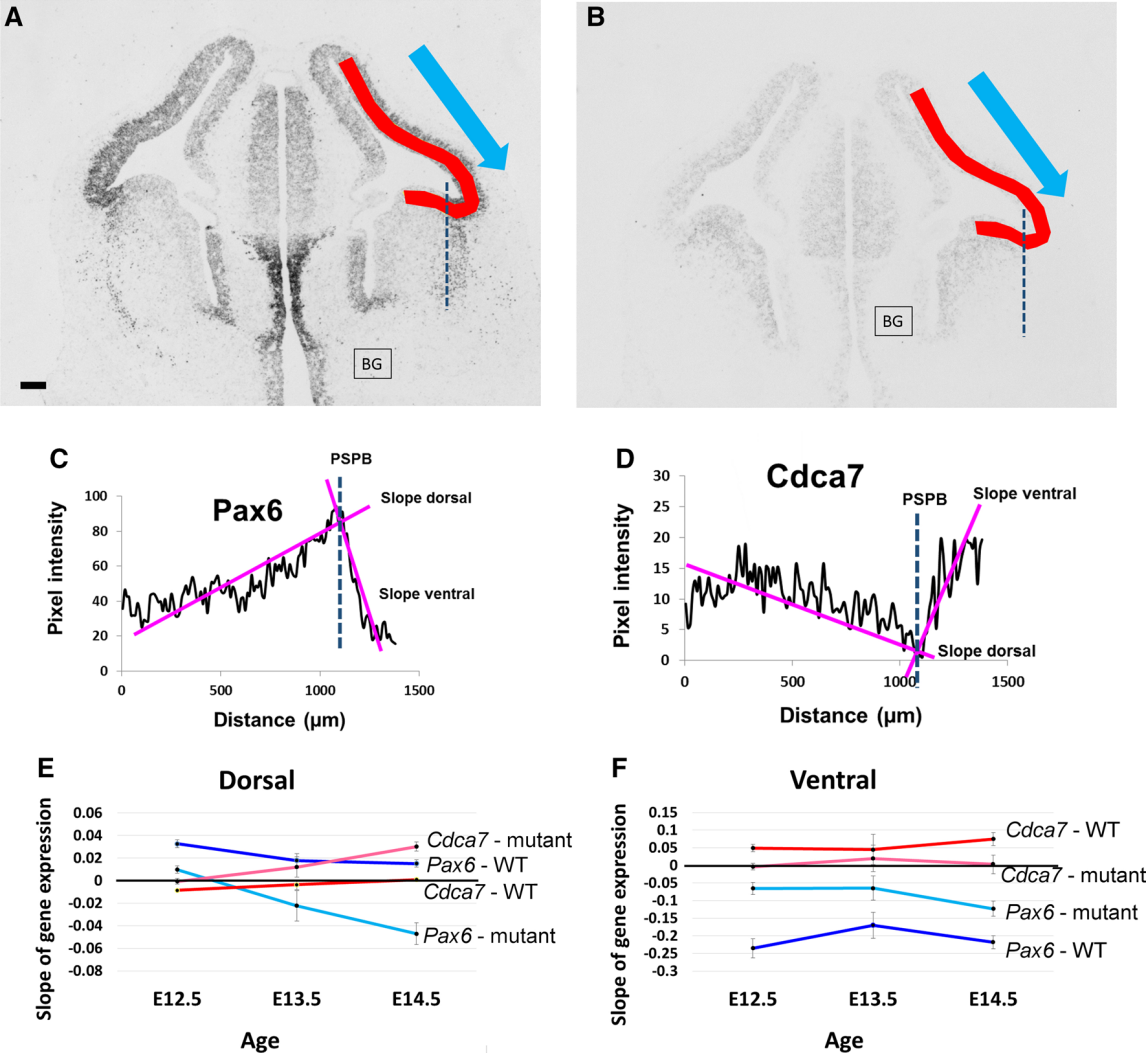
Quantitative analysis of *Pax6* and *Cdca7* expression. **A**, **B** Representative images showing the mRNA expression of (**A**) *Pax6* and (**B**) *Cdca7*. Sampling ribbons were drawn from medial cortex across the PSPB (*broken lines*) to ventral telencephalon and pixel intensities measured in the direction of the *blue arrows*. Background (BG) staining was measured in areas expressing neither gene. *Scale bar* 100 µm. **C**, **D** Average pixel intensity values along the ribbons in **A** and **B**, calculated by Fiji: the slopes of the regression lines dorsal and ventral to the PSPB were calculated. **E**, **F** Average values (±SEM) for slopes calculated as in **C**, **D** from multiple individuals (4 wild-type and *Pax6* ^−*/*−^ embryos at E12.5 and at E14.5 and 3 embryos of each genotype at E13.5)

We used a quantitative approach to confirm the breakdown in the normal expression of *Cdca7* when *Pax6* is mutated. In situ hybridizations for *Pax6* and *Cdca7* were carried out on comparable sections from wild-type and *Pax6* ^−*/*−^ littermate embryos aged E12.5, E13.5 and E14.5, mounted and processed together on the same slide to minimise batch effects. Images were analysed with Fiji software. Each image was converted to 8-bit (giving values on a greyscale of 0–255) and a ribbon whose width corresponded to that of the gene expression domains was drawn through the telencephalon from dorsal to ventral as shown in Fig. 3A, B. Average pixel intensities were calculated in 10 µm bins along the ribbon. Background staining was measured in areas expressing neither gene and average pixel intensities were corrected by subtracting BG values to obtain intensity profiles such as those shown in Fig. 3C, D. The profiles were divided into a pallial and a subpallial part based on the expression pattern of the *Pax6* gene. Regression analysis gave values for the slopes on the two sides (Fig. 3C, D). Average slopes from several animals were combined to give the graphs in Fig. 3E, F (data are from 4 wild-type and 4 *Pax6* ^−*/*−^ embryos at E12.5 and at E14.5 and 3 embryos of each genotype at E13.5).

In the pallium (i.e. dorsal to the PSPB; Fig. 3E), two way ANOVA showed significant effects of both genotype and age on the gradients of *Pax6* and *Cdca7*, with significant interaction effects for both genes (for *Pax6*: p < 0.001 for age, p < 0.001 for genotype and p = 0.017 for interaction; for *Cdca7*: p < 0.001 for age, p < 0.001 for genotype and p = 0.026 for interaction). In wild-types, the gradients of *Pax6* and *Cdca7* expression were opposite at E12.5 (Fig. 3E). Their magnitudes declined significantly over the following days, approaching (in the case of *Pax6*) or reaching (in the case of *Cdca7*) zero by E14.5 (dark blue and red lines in Fig. 3E) (E12.5 vs E14.5: for *Pax6*, p = 0.011; for *Cdca7*, p = 0.018; Tukey tests). In *Pax6* ^−*/*−^ pallium, the gradient of *Pax6* expression was much smaller than normal at E12.5 (p < 0.05, Student’s *t* test) and then reversed (light blue line in Fig. 3E). There was no gradient of *Cdca7* expression at E12.5 (a significant difference to the situation in wild-types; p < 0.05, Student’s t-test) but one appeared, with a slope opposite to normal, over the subsequent 2 days (pink line in Fig. 3E; gradients were significantly different at E14.5, p < 0.05, Student’s t-test) (E12.5 vs E14.5: for *Pax6*, p < 0.001; for *Cdca7*, p < 0.001; Tukey tests).

On the ventral side of the PSPB (Fig. 3F), two way ANOVA showed a significant effect of genotype but not age on the gradients of *Pax6* and *Cdca7*, with no significant interaction effect for either gene (for *Pax6*: p < 0.001 for age; for *Cdca7*: p = 0.015 for age). *Pax6* and *Cdca7* were expressed in opposing gradients from E12.5-14.5 (dark blue and red lines in Fig. 3F), while in *Pax6* ^−*/*−^ embryos the gradients of *Pax6* were less steep than normal (light blue line in Fig. 3F; differences were significant at p < 0.05 at all three ages, Student’s t-tests) and the gradients of *Cdca7* were abolished (pink line in Fig. 3F; differences with wild-type were significant at p < 0.05 at E12.5 and E14.5, Student’s t-tests). These quantifications support our conclusion that the *Cdca7* expression gradient is disrupted by mutation of *Pax6*.

**Fig. 4:**
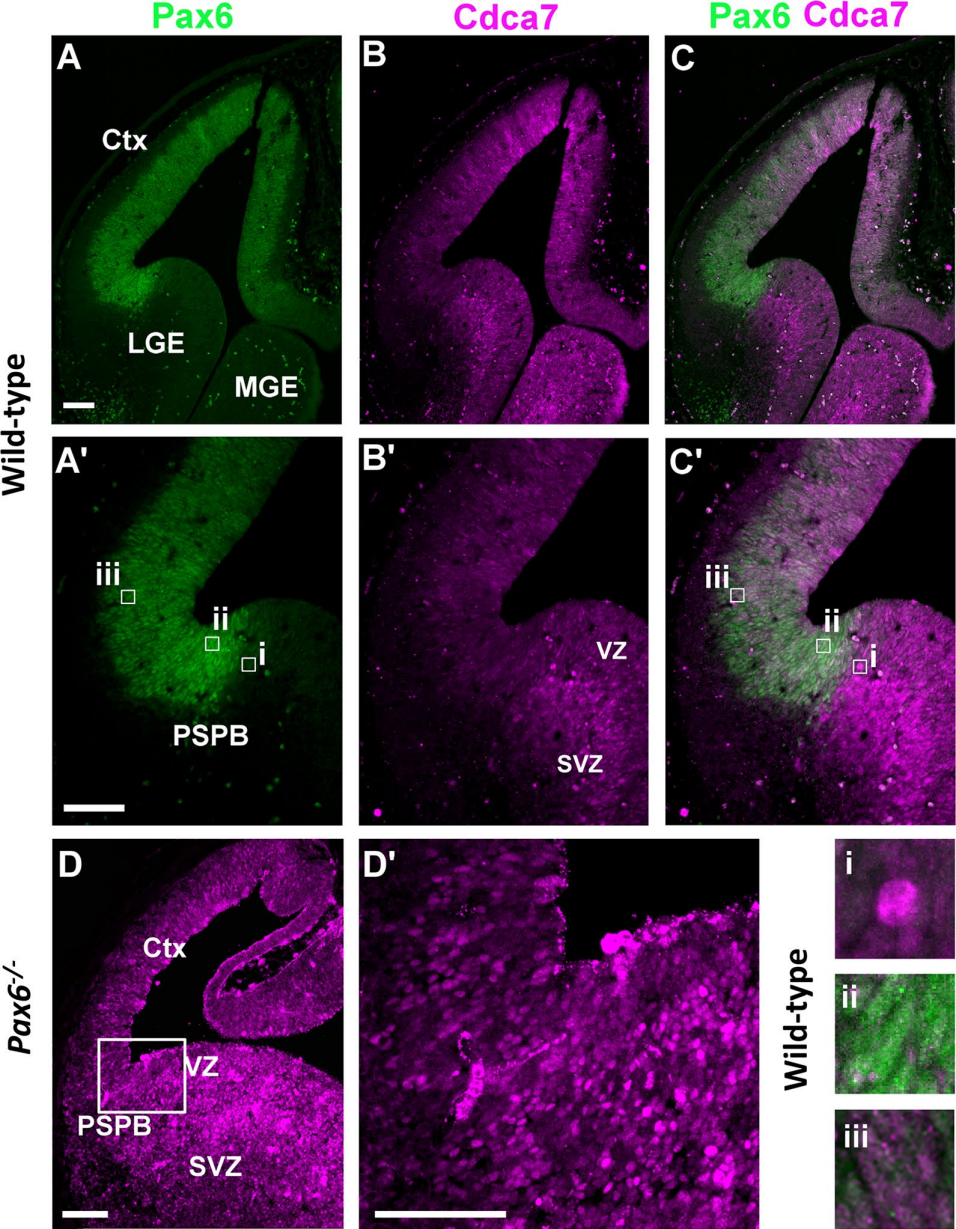
Expression of Pax6 and Cdca7 protein at E12.5. **A**–**C** Images of staining for Pax6, Cdca7 and both in the telencephalon; lateral ganglionic eminence (LGE), medial ganglionic eminence (MGE). **A**′–**C**′ Higher magnification of the regions around the PSPB in **A**–**C**. *i*–*iii* Images show the inverse relationship between the expression levels of the two proteins in individual cells. **D**, **D**′ Staining for Cdca7 in E12.5 *Pax6* ^−*/*−^ embryos; **D**′ is a higher magnification of the PSPB region in **D** (*box*). *Ctx* cortex, *VZ* ventricular zone, *SVZ* subventricular zone. *Scale bars* 100 µm

Double-immunohistochemistry for Pax6 and Cdca7 in wild-type E12.5 embryos was used to test Pax6 and Cdca7 co-expression by cells in the ventricular zone. This confirmed the complementarity of Pax6 and Cdca7 expression patterns around the PSPB (Fig. 4A, B, C, A′, B′, C′). Cells on the subpallial side of the PSPB showed low Pax6 levels and high Cdca7 levels (Fig. 4i). Interestingly, Cdca7 levels in the subpallium were particularly high in the subventricular zone (Fig. 4B′), in line with the relatively strong staining for *Cdca7* mRNA in this layer (Fig. 1E′). This strong subventricular zone staining came to an abrupt end at the PSPB (Fig. 4B′). On the pallial side of the PSPB, cells with higher levels of Pax6 staining showed lower levels of Cdca7 (Fig. 4ii). In the region further away from the PSPB, cells with higher levels of Cdca7 showed lower Pax6 staining (Fig. 4iii). Immunohistochemistry on *Pax6* ^−*/*−^ embryos showed the elevation of Cdca7 protein levels in lateral cortex and the loss of the medial to lateral gradient in its expression (Fig. 4D).

Our new findings, together with previous microarray and quantitative PCR data showing that levels of *Cdca7* increase in the pallium of *Pax6* ^−*/*−^ mutant embryos [11], indicate that high levels of Pax6 normally present in the lateral cortex repress *Cdca7* expression in this region. We next assessed the functional importance of this repression by asking what would happen if Cdca7 levels were to increase in the lateral cortex of normal embryos.

### The consequences of elevating Cdca7 expression in lateral cortex

**Fig. 5:**
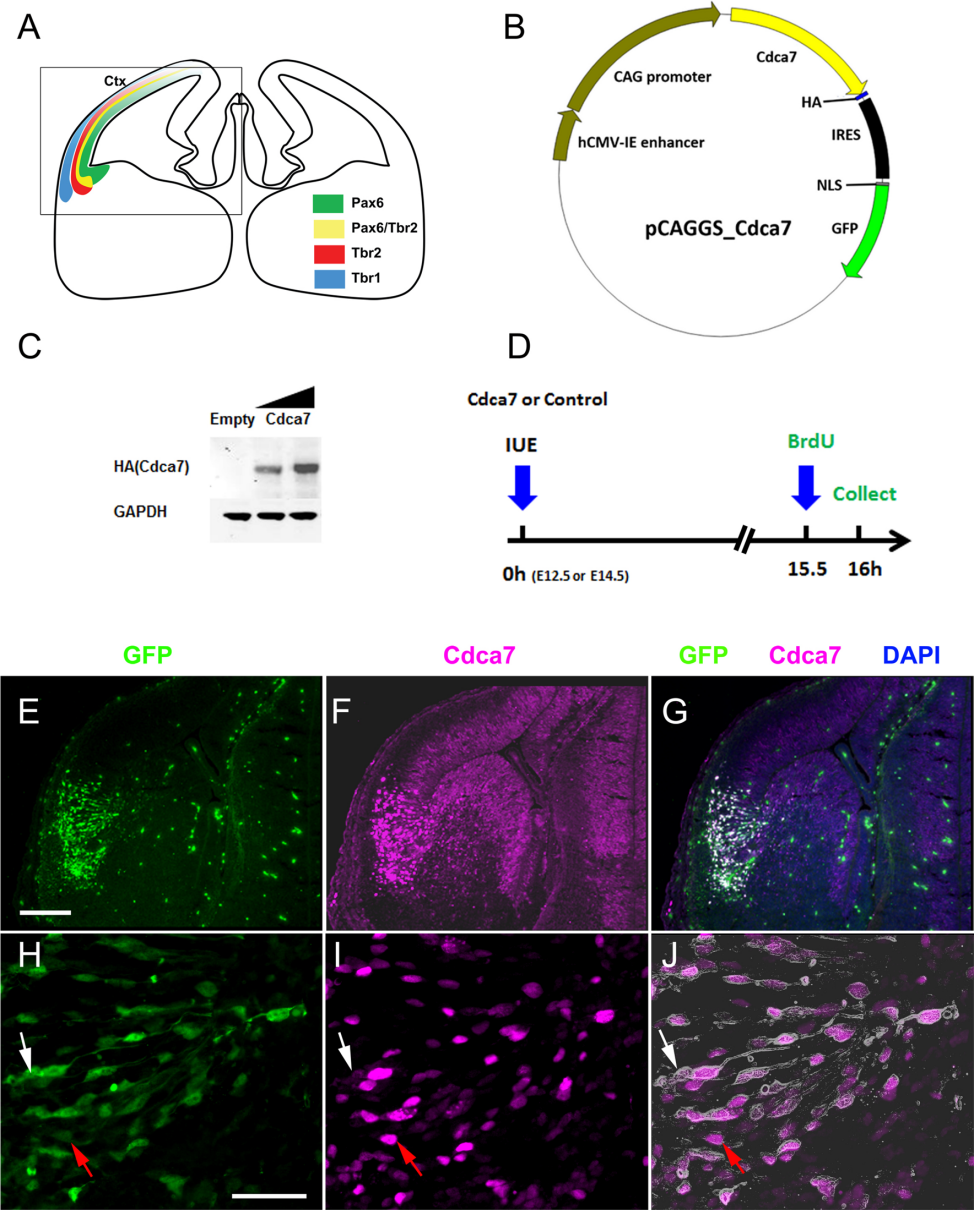
Effects of forced expression of Cdca7 in vivo. **A** Schematic showing the expression patterns of Pax6 (in radial glial cells), Tbr2 (in intermediate progenitor cells) and Tbr1 (in postmitotic neurons). Area in *box* is shown in **E**–**G**. **B** C*dca7* expression vector pCAGGS_Cdca7 containing *Cdca7* open reading frame. **C** Cdca7 expression plasmid was transiently transfected into HEK293 cells. Cell lysates were collected 48 h post transfection and Cdca7 expression was verified using an antibody against the HA epitope. The molecular weight of Cdca7 was approximately 43 kDa. GAPDH was used as a loading control. **D** Control (pCAGGS_GFP) or Cdca7 expression vector was used for in utero electroporation at E12.5 or E14.5. BrdU was injected 30 min before termination. **E**–**J** Immunofluorescence images show the expression of GFP and Cdca7 in the electroporated lateral cortex. **H**–**J** Almost all GFP+ cells co-express Cdca7 at levels obviously higher than endogenous. Outlines of GFP+ cells are overlaid on Cdca7 cells in **J**. *White arrows* indicate a cell with strong GFP but Cdca7 levels that are not obviously raised above endogenous. *Red arrows* indicate a cell with a low GFP level but a high Cdca7 level. *Scale bars* **E**–**G** 200 µm; **H**–**J** 50 µm

As cells exit the E12.5 Pax6+ cortical ventricular zone, many of them upregulate Tbr2 and become intermediate progenitors in the subventricular zone before dividing again to generate Tbr1+ neurons that enter the cortical plate (Fig. 5A) [4, 10]. Interestingly, this process is most advanced in the lateral cortex, where Cdca7 levels are most strongly suppressed by Pax6. To test whether low levels of Cdca7 in the lateral cortex are important for cortical development to proceed normally in this region, we elevated Cdca7 levels in progenitors in the lateral cortex of normal embryos and examined the effects on cell proliferation and differentiation into Tbr2+ and Tbr1+ cells.

The open reading frame of a full length *Cdca7* cDNA was tagged with influenza hemagglutinin (HA) sequence to generate an HA epitope-tagged version of Cdca7. This was cloned into the expression vector pCAGGS_GFP, which expresses green fluorescent protein (GFP) tagged with a nuclear localization signal (NLS) (Fig. 5B). The Cdca7 expression plasmid was validated by transient transfection of HEK293 cells. At 48 h post transfection, cell lysates were analysed by western blot with an antibody against the HA epitope (Fig. 5C). The level of expression of Cdca7 correlated with the concentration of vector used in the transfection. The lateral cortex of wild-type E12.5 or E14.5 embryos was electroporated in utero with either the Cdca7 expression plasmid pCAGGS_Cdca7 or empty vector (pCAGGS_GFP) as a control. Embryos were injected with bromodeoxyuridine (BrdU) 15.5 h later and culled after a further 0.5 h (Fig. 5D). GFP+ cells were detected through the depth of the lateral cortex 16 h post-electroporation (Fig. 5E). Immunohistochemistry for Cdca7 showed that they expressed higher levels of Cdca7 than their neighbours (Fig. 5F, G). Since the Cdca7 antibody gave clear evidence of increased expression, confirmation using the HA tag in vivo was not considered necessary. We examined 625 GFP+ cells randomly selected from six different sections through the electroporated areas of these embryos and found that 98.5% of them showed Cdca7 expression increased above endogenous levels (Fig. 5H–J; these images illustrate a rare example of a GFP+ cell that did not appear to have elevated Cdca7, white arrows). In general, staining for Cdca7 was stronger in cells with stronger GFP signals, but there were frequent exceptions (e.g. red arrows in Fig. 5H–J show an example of a cell with high Cdca7 but low GFP). Most likely this was due to the use of an internal ribosomal entry site (IRES) in the construct. It is known that IRES activities can vary greatly between cells due to intercellular variation in the levels of positive or negative regulatory IRES *trans*-acting factors that influence IRES function but not cap-mediated initiation [35].

We estimated the level of Cdca7 expression from the electroporated cells relative to surrounding non-electroporated cells by randomly selecting 30 GFP-expressing cells and 30 intermingled GFP-non-expressing cells within the lateral cortex, outlining each cell and measuring the intensity of the Cdca7 signal from each using Fiji software. We found that the average intensity of the Cdca7 signal was 4.4-fold higher in the GFP+ than in the GFP− cells. To put this difference into context, we then carried out the same analysis with 30 GFP-non-expressing cells from the ventral telencephalon, where Cdca7 is highly expressed by some cells; in this case we selected cells with high levels of expression, located in the ventral telencephalic subventricular zone (Fig. 4B′). We found that the average intensity of the Cdca7 signal from these high-expressing ventral cells was 3.9-fold higher than that from the non-electroporated lateral cortical cells. These values suggest that the electroporated cells in the lateral cortex achieved levels of Cdca7 that were similar to the endogenous levels present in the most highly expressing cells in the ventral telencephalon. This approximates to the situation in *Pax6* ^−*/*−^ mutants (Fig. 4D, D′), where the intensity of Cdca7 signals from lateral telencephalic cells becomes closer to that from ventral telencephalic cells (measurements done as above showed an average intensity of Cdca7 signal in ventral telencephalic cells only 1.8-fold above that of lateral cortical cells).

**Fig. 6:**
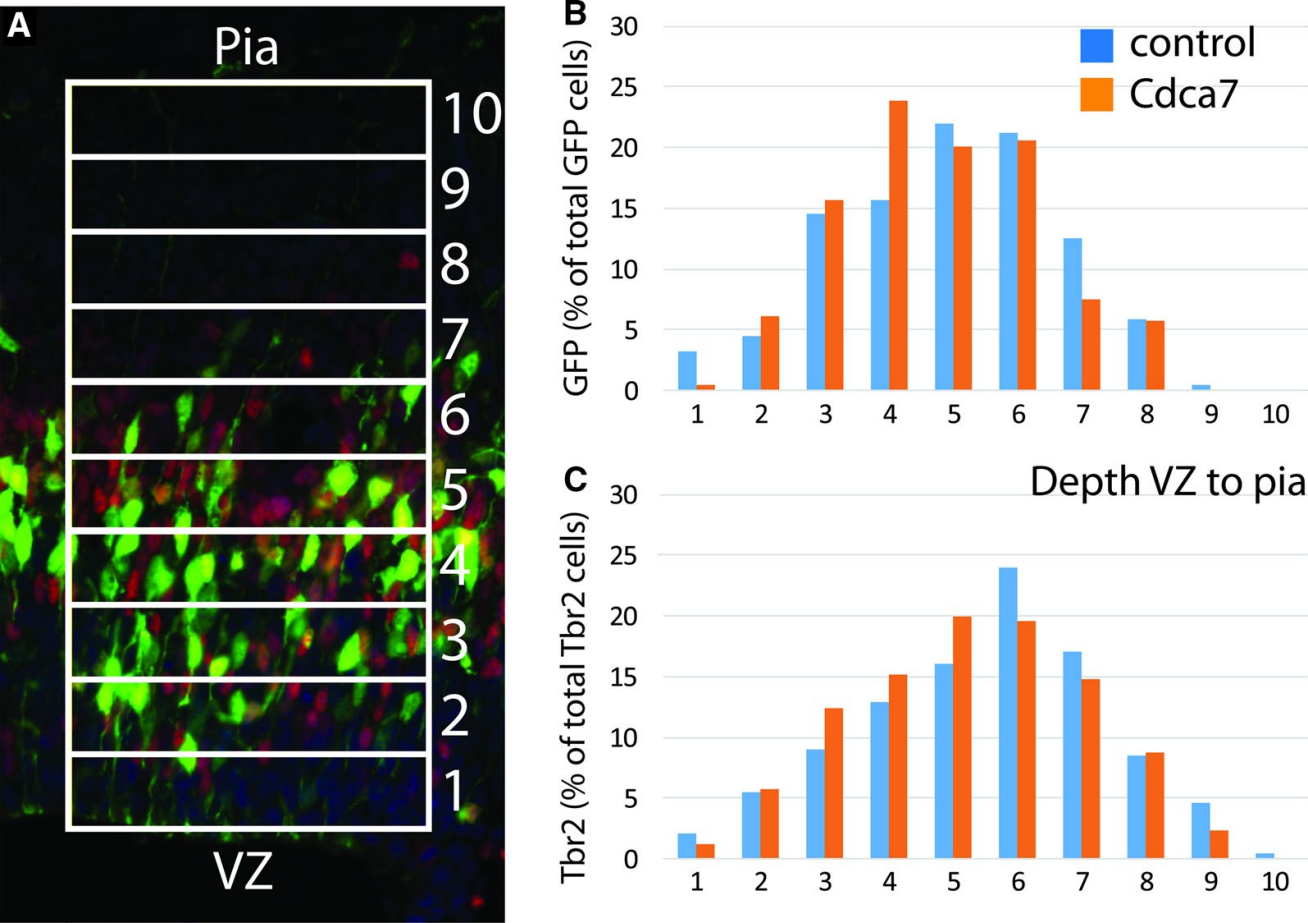
The distribution of electroporated cells with raised Cdca7 levels in lateral cortex is not different from control. **A** GFP+ (*green*) and Tbr2+ (*red*) cells were counted in ten 250 µm-wide bins of equal depth from ventricular zone to pia (counts were from three E12.5+16h embryos electroporated with control and three E12.5+16h embryos electroporated with Cdca7-expressing constructs). **B**, **C** Average proportions of GFP+ and Tbr2+ cells in each bin are plotted. The average depth of GFP+ cells on the scale of 1–10 was 4.95 ± 0.42 (SEM) in control and 4.78 ± 0.31 in experimental animals (ns, p = 0.76, Student’s t-test). The average depth of Tbr2+ cells was 5.48 ± 0.39 in control and 5.23 ± 0.22 in experimental animals (ns, p = 0.60, Student’s t-test)

**Fig. 7:**
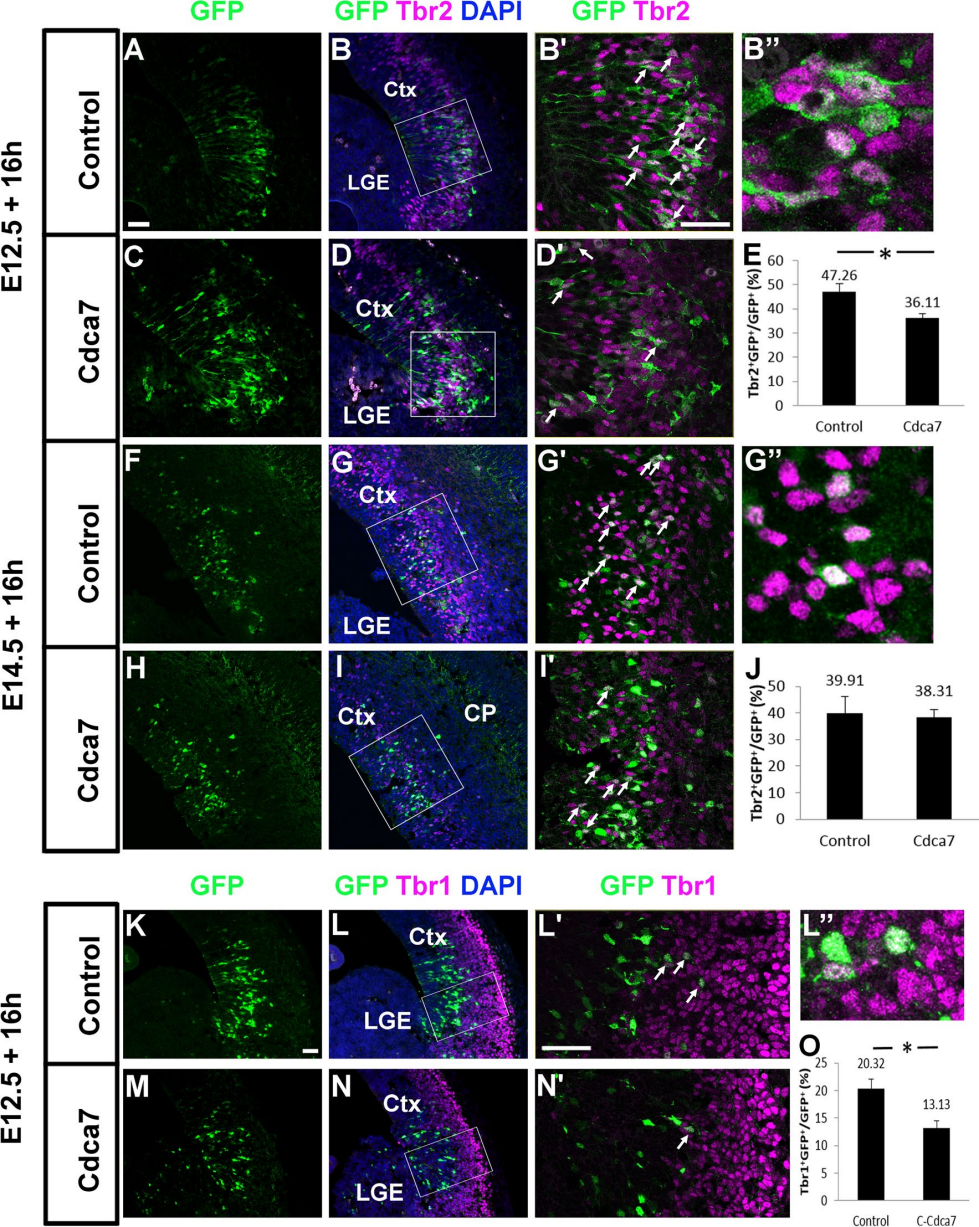
Raising Cdca7 levels in E12.5 lateral cortex affects the production of Tbr2- and Tbr1-expressing cells. Brains electroporated with either the control or Cdca7 plasmid at **A**–**D**, **K**–**N** E12.5 and **F**–**I** E14.5 reacted for **A**–**D**, **F**–**I** Tbr2 or **K**–**N** Tbr1. **B**′, **D**′, **G**′, **I**′, **L**′, **N**′ High magnification of the areas outlined in **B**, **D**, **G**, **I**, **L**, **N**. *Arrows point* to cells co-labelled with Tbr2/Tbr1 and GFP and **B**″, **G**″ and **L**″ show magnified examples. **E** The frequencies of Tbr2+ cells in the population of GFP+ cells after E12.5 electroporation (mean ± SEM; control, n = 4: Cdca7, n = 3): the proportion in the Cdca7 group is significantly lower than that in the control group (p < 0.05, Student’s t-test). **J** The frequencies of Tbr2+ cells in the population of GFP+ cells after E14.5 electroporation (mean ± SEM; control, n = 3: Cdca7, n = 4): there is no significant difference (Student’s t-test). **O** The frequencies of Tbr1+ cells in the population of GFP+ cells after E12.5 electroporation (mean ± SEM; control, n = 4: Cdca7, n = 3): the proportion in the Cdca7 group is significantly lower than that in the control group (p < 0.05, Student’s t-test). All *scale bars* 50 µm

**Fig. 8:**
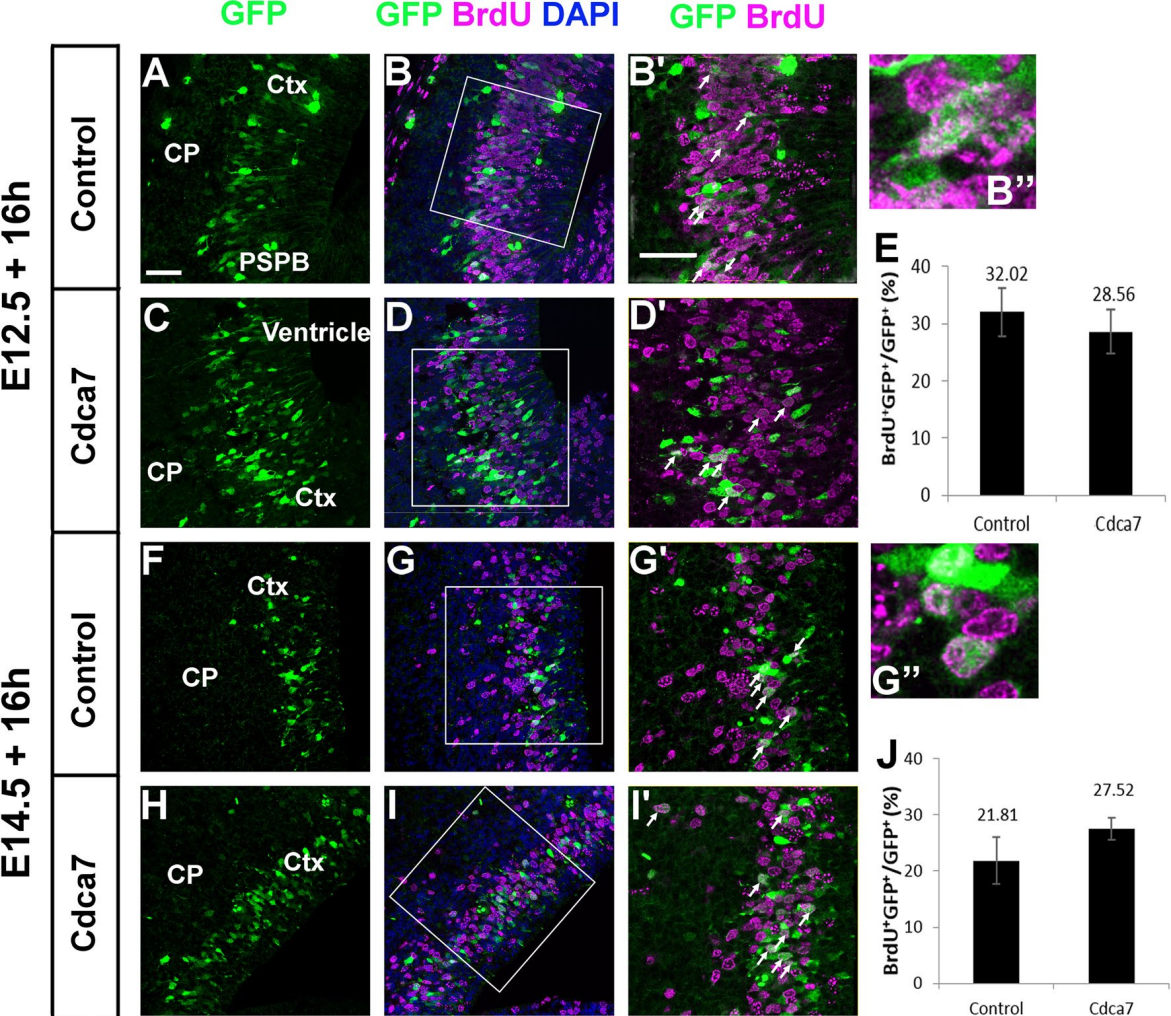
Raising Cdca7 levels in lateral cortex has no detectable effects on cell proliferation. **A**–**D**, **F**–**I** Brains electroporated with either the control or Cdca7 plasmid at **A**–**D** E12.5 and **F**–**I** E14.5 processed for BrdU incorporation. **B**′, **D**′, **G**′, **I**′ High magnification of the areas outlined in **B**, **D**, **G**, **I**. *Arrows point* to cells co-labelled with BrdU and GFP and **B**″ and **G**″ show magnified examples. **E** The frequencies of BrdU+ cells in the population of GFP+ cells after E12.5 electroporation (mean ± SEM; control, n = 4: Cdca7, n = 3): there is no significant difference (Student’s t-test). **J** The frequencies of BrdU+ cells in the population of GFP+ cells after E14.5 electroporation (mean ± SEM; control, n = 3; Cdca7, n = 4): there is no significant difference (Student’s t-test). *CP* cortical plate. All *scale bars* 50 µm

We examined all GFP+ electroporated cells 16 h after electroporation at either E12.5 or E14.5 in a series of sections stained for: GFP and Tbr2; GFP and Tbr1; GFP and BrdU. We found no evidence that the elevation of Cdca7 levels altered the distribution of affected cells through the cortical depth 16 h after electroporation (Fig. 6). Average proportions of GFP+ cells that were double-labelled for Tbr2, Tbr1 or BrdU were obtained from an analysis of multiple electroporated embryos (Figs. 7, 8).

There was a significant reduction by ~20–25% in the proportion of GFP+ cells that were Tbr2+ in embryos electroporated with Cdca7 at E12.5 (Fig. 7A–E), although not in those electroporated at E14.5 (Fig. 7F–J). In both cases, the GFP+ Tbr2+ cells were in the same layer as the non-electroporated (GFP−) Tbr2+ cells. These data suggest that raised levels of Cdca7 reduce the generation of Tbr2+ intermediate progenitors in the early stages of corticogenesis. Early-generated Tbr2+ progenitors are known to produce Tbr1+ neurons [4] and, 16 h after electroporation on E12.5, small numbers of GFP+ Tbr1+ cells had reached the deep edge of the cortical plate. The proportions of GFP+ cells that were Tbr1+ were significantly lower in cells overexpressing Cdca7 than in control cells Fig. 7K–O, which is likely to be a consequence of the underproduction of Tbr2+ intermediate progenitors.

Analysis of the proportion of cells labelled by BrdU shortly before animals were sacrificed allowed us to assess whether elevated Cdca7 levels in the lateral cortex had a drastic effect on proliferation, for example bringing effected cells out of the cell cycle. We found that 20–30% of GFP+ cells took up BrdU in both control and experimental groups following electroporation at E12.5 or E14.5 (Fig. 8). At neither age did electroporation with the Cdca7-expressing plasmid have a significant effect on the proportions of GFP+ cells that were in S-phase in the lateral cortex 16 h later (Fig. 8).

Our findings indicate that the main effect of elevated Cdca7 expression in early embryonic lateral cortical progenitors is to reduce the production of cells expressing Tbr2, which is the hallmark of intermediate progenitors.

## Discussion

Based on new findings reported here, we suggest that one of the actions of Pax6 in the embryonic cerebral cortex is to suppress Cdca7 expression and thereby enhance the production of Tbr2+ intermediate progenitors. Interestingly, our experiments suggested that this suppressive effect is most prominent in the lateral cortex early in corticogenesis, where cortical levels of Pax6 expression are highest [11]. Since Tbr2+ intermediate progenitors are transit amplifying cells, an increase in their numbers will increase neuronal output [23–25]. It is well known that lateral cortex develops ahead of other cortical regions: for example, at early stages lateral cortex is thicker, has many more Tbr2+ intermediate progenitors and more neurons in its cortical plate than medial regions. Previous studies have shown that one of the striking anatomical abnormalities of the *Pax6* ^−*/*−^ forebrain is a failure of the lateral cortex to expand ahead of more medial regions. This regional defect has been linked to a disproportionate underproduction of Tbr2+ cells in the lateral cortex of *Pax6* ^−*/*−^ embryos, a loss which equilibrates the numbers of Tbr2+ cells across all cortical regions [12]. It is possible that, in normal development, suppression of Cdca7 by high levels of Pax6 is an important factor allowing lateral cortex to develop ahead of other cortical regions.

On the ventral side of the PSPB in wild-type embryos, Pax6 levels are lower and Cdca7 levels are higher, particularly in the subventricular zone of the subpallium. As an extension of the present study, it would be interesting to test with gene knock-out or knock-down experiments whether the particularly high levels of Cdca7 that we observed in the subpallial subventricular zone contribute to the lack of Tbr2 expression in the cells of this region.

Relatively little is known about the mechanisms of action of Cdca7. Human and mouse CDCA7/Cdca7 are nuclear proteins which contain a zinc finger domain in the C-terminus [13, 15]. In humans, *CDCA7* was first identified as a MYC direct target and CDCA7 protein is often found to be overexpressed in human solid tumours [16, 26–28]. Previous research has indicated an interaction of Cdca7 with Notch, a molecule with actions in cell fate determination that is expressed in many tissues including the cortex [29]. The evidence for this interaction comes from studies on a completely different cell type, namely E11.5 aorta-gonad-mesonephros tissue. These studies suggest that Cdca7 is a direct, positively regulated target of Notch signalling [14]. In the developing cortex, cells that receive Notch signalling preserve their radial glial identity [30, 31]. It is possible, therefore, that Cdca7 acts downstream of Notch activation to enhance the production of cells with radial glial identity, which might explain why Cdca7 overexpression reduced the proportions of cells acquiring the Tbr2+ intermediate progenitor cell fate in our experiments. Further experiments might address whether, in lateral cortex, Notch activation affects *Cdca7* expression and whether the repressive effects of Pax6 on Cdca7 expression are direct, or an indirect consequence of effects on Notch signalling, or both.

Cdca7 overexpression early in corticogenesis reduced not only the production of Tbr2+ intermediate progenitors but also the production of Tbr1+ cells. The divisions of Tbr2+ cells generate Tbr1+ neuronal precursors early in corticogenesis. The length of cell cycles in the cerebral cortex is around 10 h at E12.5, which is a few hours shorter than the 16 h between electroporation and sacrifice in our experiments [32, 33]. It seems likely, therefore, that the expression plasmid entered some cells that had already started to move to the subventricular zone, where they underwent a further division to produce Tbr1+ neuronal precursors. In the subventricular zone, approximate 25% of Tbr2+ cell co-express Tbr1 [34], presumably as they transform from their progenitor to their neuronal precursor state during or following division. It is plausible, therefore, that the reduction of Tbr1+ cells following Cdca7 overexpression is a consequence of the reduction of Tbr2+ intermediate progenitors. Further work is required to test this possibility, as well the additional hypothesis that some of the reduction of Tbr1+ cells results from reduced direct neurogenesis from radial glial cells.

Finally, our quantitative analysis of *Pax6* and *Cdca7* mRNA cortical expression gradients following mutation of the *Pax6* gene indicated that both become highly abnormal from E12.5 to E14.5. By E14.5, the slopes of both gradients had reversed from normal. This result highlights the complexity of the molecular interactions that establish graded expression patterns. It indicates that Pax6 is not the only factor that can affect differential *Cdca7* expression levels across the developing cortex. Changes in the expression of *Pax6* mRNA in *Pax6* ^−*/*−^ mice are likely to result from a loss of direct or indirect autoregulation of the *Pax6* locus, which is known to be a prominent element in the control of expression of the *Pax6* gene [36].

## Conclusions

In summary, this study provides evidence of counter-gradients of Pax6 and Cdca7 during embryonic ages E12.5 to E14.5 in mice and this relationship is disrupted when Pax6 is absent. In addition, elevated Cdca7 in the lateral cortex reduces the production of intermediate progenitors and postmitotic neurons, suggesting that repression of Cdca7 by Pax6 is required for the normal production of intermediate progenitors in this region.

## Abbreviations

Cdca7: cell division cycle associated 7
AC: anterior cortex
BG: background
BrdU: 5-bromo-2′-deoxyuridine
CP: cortical plate
Ctx: cortex
DAPI: 4′,6-diamidino-2-phenylindole
DIG: digoxigenin
GAPDH: glyceraldehyde 3-phosphate dehydrogenase
GFP: green fluorescent protein
HA: hemagglutinin
HEK: human embryonic kidney
LC: lateral cortex
LGE: lateral ganglionic eminence
MGE: medial ganglionic eminence
NLS: nuclear localization signal
ORF: open reading frame
pCAGGS_GFP: pCAGGS_IRES-NLS-GFP
PSPB: pallial-subpallial boundary
SEM: standard error of the mean
SVZ: subventricular zone
VZ: ventricular zone
